# Surface EMG-Validated Multi-DoF Wheelchair-Based Rehabilitation Device

**DOI:** 10.3390/bioengineering13030350

**Published:** 2026-03-18

**Authors:** Jagan P, Madhav Rao

**Affiliations:** Electronics & Communication Department, International Institute of Information Technology-Bangalore, Bengaluru 560100, India; mr@iiitb.ac.in

**Keywords:** surface EMG, post-stroke, rehabilitation, upper and lower limb, joint angle, Degrees of Freedom (DoF), actuators

## Abstract

Rehabilitation is a critical component in the recovery of patients with either complete or partial loss of motor movements. Repeated and slow limb movements are usually advised by practitioners. Advanced robotic systems can help to configure monotonous movements and accelerate the recovery process as an alternative to therapist-assisted motions, especially during the later phase of recovery. In this work, robotic-assisted human limb movements are engineered and augmented with a novel electromyography (EMG) signal to characterize the movements. The proposed lower- and upper-limb assistive system is designed on a wheelchair platform and is IoT-enabled. The proposed assistive system is designed for patients affected with hemiplegia, paraplegia and tetraplegia. Existing state-of-the-art (SOTA) systems are typically focused on either the upper or lower limbs, with limited degrees of freedom (DoF). The IoT framework for remote access enables the possibility of home-based rehabilitation. A prototype was successfully developed and experiments to characterize various muscle movements using the proposed system were performed.

## 1. Introduction

Stroke stands second among the leading causes of mortality and ranks third for morbidity, thereby impacting the quality of life of millions of individuals and their families around the world [1,2,3]. Global stroke-related medical costs are projected to be USD 94.3 billion in 2035 [4]. Its impact is particularly severe in low- and middle-income countries, where limited access to medical facilities and rehabilitation support contributes to a higher prevalence of stroke-related disabilities [3]. The growing number of stroke patients is placing a significant burden on therapists and is exacerbated by a steady decline in the therapist–patient ratio [5]. This imbalance often leads to compromised care quality, delays in recovery, and fatal outcomes. The shortage of healthcare professionals highlights the urgent need for reliable home-based rehabilitation solutions that can be programmed once and reused consistently throughout the recovery period. The history of muscle pain and dysfunction is understood through a four-factor model—tissue, emotional, movement and postural factors [6,7]. Physiotherapy exercises typically focus on repetitive movements of the upper and lower limbs to promote muscle recovery. In this context, robotic systems offer a promising solution by automating these movements and enabling continuous and effective limb actuation, such as through intensive therapy and precise movement, to support and accelerate the rehabilitation process beyond traditional methods [8]. The potential benefits of rehabilitation robots include personalized therapy tailored to individual needs, continuous progress monitoring and a reduction in hospital visits. However, key challenges persist, such as high implementation costs, patient acceptance and adaptability for smooth integration into existing healthcare systems.

The integration of IoT-enabled exoskeletons or robots provides a significant advantage by enabling experts to remotely monitor rehabilitation sessions, while allowing patients to perform exercises at home with support from a family caregiver. Data is collected during rehabilitation sessions and transported to the cloud to be securely stored and analyzed. This facilitates real-time remote monitoring and assessment by medical experts. The role of robotics in the rehabilitation process has been extensively explored in the literature [9]. For instance, movements such as flexion and extension of the four fingers, wrist and elbow, as well as the pinch motion between the thumb and index finger, are thoroughly examined in [10]. A system for upper-limb rehabilitation that utilizes a Permanent Magnet Alternating Current (PMAC) motor to drive a flexible wire-pulling mechanism, enabling three degrees of freedom (3DoF) in the wrist and flexion–extension movement in the elbow, is presented in [11]. A system for remotely monitoring elbow joint angles enables physicians to track movement in real time via a graphical user interface. This system, which collects data over Wi-Fi, combining inertial signals from an MPU6050 sensor—featuring a three-axis gyroscope and accelerometer—with EMG signals from the biceps brachii and triceps brachii muscles, is evaluated in [12]. A coordinated lower-limb method in which EMG signals generated from the upper limbs are used to activate a knee actuation system in the lower limbs is described in [13]. The development of a 5DoF upper-limb system that supports an individual’s wrist, elbow and shoulder to aid object grasping with the right hand and allows the system to be controlled with the other hand is presented in [14]. The fabrication of an eight-channel surface EMG acquisition system, designed to capture muscle signals associated with finger and wrist movements, which are analyzed using a Support Vector Machine (SVM) training model to classify and interpret the signal characteristics for precise control of finger and wrist actuation based on muscle activity patterns, is discussed in [15]. An integrated hardware–software system for sEMG acquisition, processing and analysis has been developed that employs CNNs for pattern recognition. The proposed approach enhances signal quality, mitigates cross-talk and addresses the non-stationary nature of sEMG recordings [16]. Surface-EMG-based rehabilitation data for the wrist, shoulder and leg is presented in [17]. A device designed to prevent Deep Venous Thrombosis (DVT) in the lower limbs, aimed at reduce blood clots by improving blood circulation in the legs using a linear stepper motor in combination with a rubber cuff placed around the calf muscle, with an air compressor enabling cyclical inflation and deflation of the cuff, is presented in [18]. A 3D-printed wearable device for upper-limb anthropomimetic rehabilitation with a DC motor interfaced for finger and wrist actuation, along with analysis of its performance characteristics, is described in [19]. Ref. [20] presents an integrated upper- and lower-limb system. However, it is not IoT-enabled, nor does it have 3DoF in the wrist and hip movement. The Finite Element Method (FEM) is essential in robotic rehabilitation in order to analyze stress distribution, deformation and fatigue under repetitive loading to ensure structural safety, durability and optimized lightweight design for patient comfort [21]. IoT offers clear clinical benefits in rehabilitation by enabling continuous monitoring of patient progress in line with individual needs through real-time data and facilitating early detection of potential health issues. However, its implementation also raises challenges regarding cybersecurity, patient privacy and ensuring reliable data transmission for accurate decision-making. The system can incorporate encrypted data transmission to ensure reliable and secure home rehabilitation with continuous operation for real-time patient feedback. Redundant sensor measurements and periodic self-diagnostics detect anomalies [22]. Augmented Reality (AR) and Virtual Reality (VR) are increasingly used in rehabilitation to create immersive, interactive and motivating environments for patients [23]. Table 1 shows a summary of the survey of the literature. It can be observed that most of the systems discussed are IoT-enabled or wheelchair-based. Wheelchair-based systems give extra portability and flexibility in patients’ movement. Our proposed research aims to develop an integrated rehabilitation device for both upper and lower limbs, with validation conducted through surface electromyography (sEMG) analysis. Section 2 presents the system model, Section 3 details the upper-limb exoskeleton design, Section 4 covers surface EMG signals, Section 5 describes the experiments and results, and Section 6 concludes the study.

## 2. System Model

**Figure 1:**
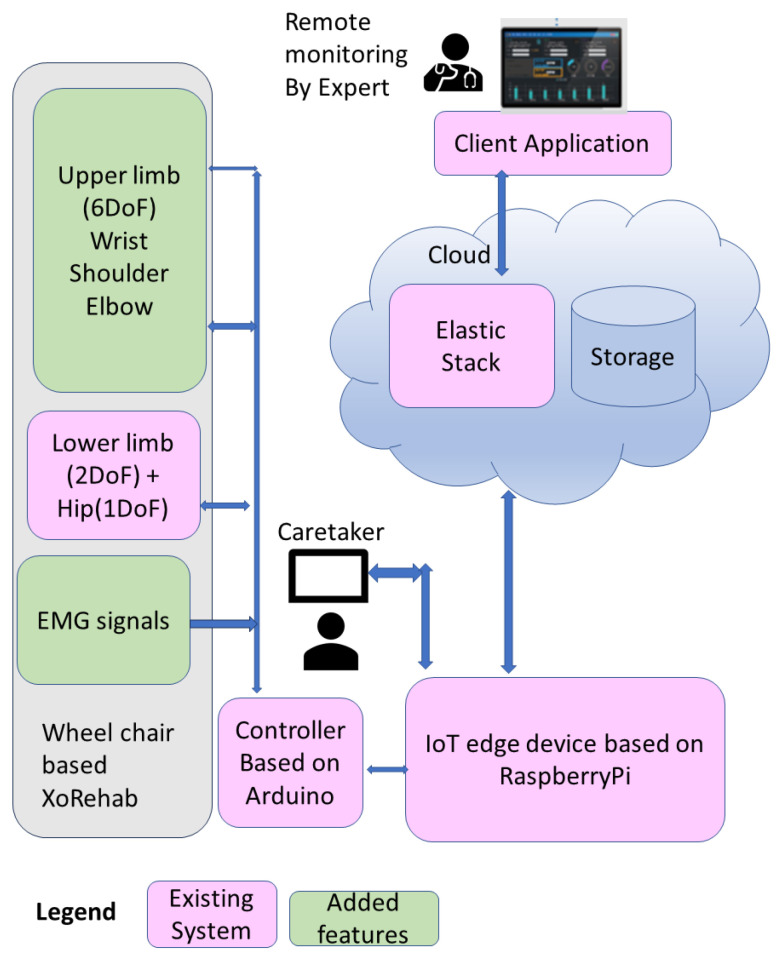
Proposed system architecture.

The proposed system model, shown in Figure 1, is an upper- and lower-limb rehabilitation structure built upon a wheelchair to provide portable access and comfort to patients while undergoing routine therapy. Typical rehabilitation therapy involves a sequence of motions in the wrist, elbow, shoulder, hip, knee and ankle joints based on the patient’s limb injuries. The proposed system offers no need for repositioning the patient during upper- and lower-limb routines, which was found to be lacking in most of the modular SOTA forms. The device presents 9DoF, which includes 6DoF in the upper limbs and 3DoF in the lower limbs (including a backrest mechanism). The upper-limb rehabilitation focuses on 3DoF in the wrist for flexion–extension, abduction–adduction and radial–ulnar deviation. The elbow provides 1DoF towards flexion–extension and 2DoF for the shoulder, consisting of flexion–extension and abduction–adduction. The lower-limb rehabilitation device focuses on 1DoF for the knee, ankle and hip joints, each achieving flexion–extension movements. The backrest mechanism assisting the hip joint bends backwards, which makes the seat-based wheelchair into a stretcher, ensuring the relaxation of the patient during therapy in this recliner position. The lower-limb rehabilitation system’s design and its structural development, equipped with an IOT mechanism using ELK, is discussed elaborately in [24]. The upper-limb actuation system is discussed elaborately in the subsequent sections.

## 3. Upper-Limb Exoskeleton Design

**Figure 2:**
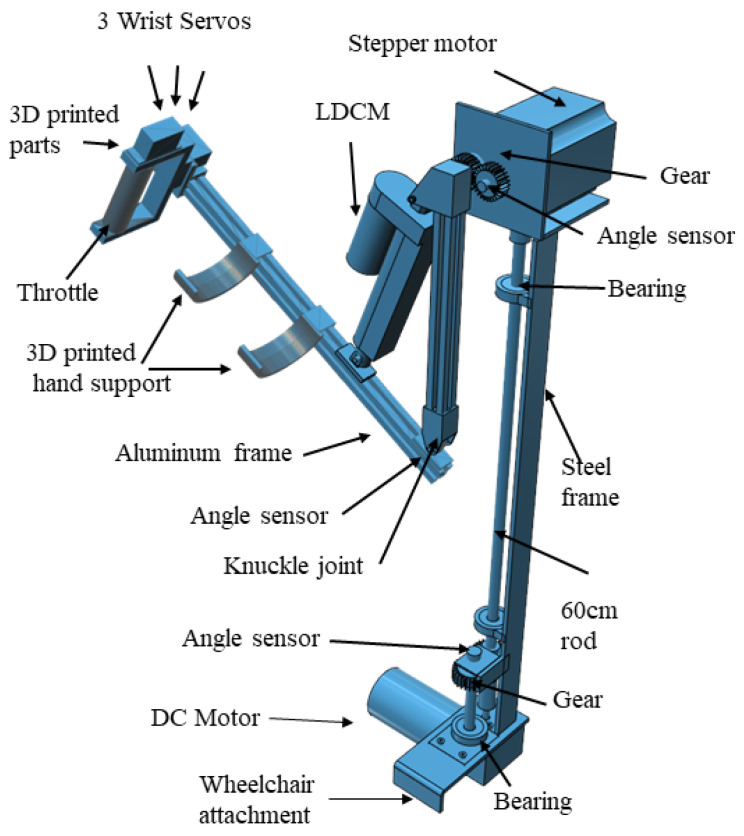
The 3D design of the upper-limb mechanism with the wrist, elbow and shoulder.

The proposed assistive upper-limb system is built on an Armeo spring to offer 6DoF. The Armeo spring is attached on either sides of the wheelchair. The upper-limb mechanism covers three regions of operations, including the wrist, elbow and shoulder joints. The Armeo spring is configured to maintain high strength at the base and ensure the end is lightweight, in order to reduce motor torque and cost. The design employs three different materials to provide the required strength. The shoulder region is supported with a steel frame, while the elbow region is further covered with an aluminum frame, and the wrist region is constructed with 3D-printed parts using Poly Lactic Acid (PLA) material. The 3D design of the upper-limb mechanism is shown in Figure 2.

### 3.1. Wrist Design

A maximum flexion movement of 38°and extension movement of 40°, for a total of 78°, is possible. Similarly, a total angular pronation and supination movement of 180° is expected for a healthy wrist joint. Further, the human wrist joint has the ability to perform 15° of radial deviation and 45° of Ulnar deviation according to human physiology and anatomy [25]. Hence, to accomplish this specified complete range of motion, the wrist mechanism is built with three servo motors, accomplishing 3DoF. The servo rating of 35 kg/cm stall torque, with a maximum voltage of 8 V, is employed to provide controlled servo angular movement. The Armeo spring for the wrist is built based on a throttle-based actuation system for exerting flexion–extension movements. The throttle model is connected to the shaft of servo motor #1 and the other servo motor is attached on top of servo motor #2 to control the abduction–adduction actions. The materials used to hold the servo motors are completely formed using PLA via 3D printers with 70% infills to retain the lightweight factor and subsequently offer more strength.

### 3.2. Elbow Design

The range of motion for the elbow joint is extendable to 142° according to human physiology and anatomy [26]. This motion activates the triceps brachii and brachioradialis muscles. A Linear Direct Current Motor (LDCM) of 12 V with a stroke length of 10 cm is employed to actuate the elbow joint, and connected to aluminum enclosures covering the shoulder joint & wrist joints. The flexion and extension motions of the elbow joint are made possible through this design, which is further equipped with an angle feedback rotatory sensor. An elbow enclosure is built using an aluminum frame to hold the LDCM and the wrist mechanism.

### 3.3. Shoulder Design

**Figure 3:**
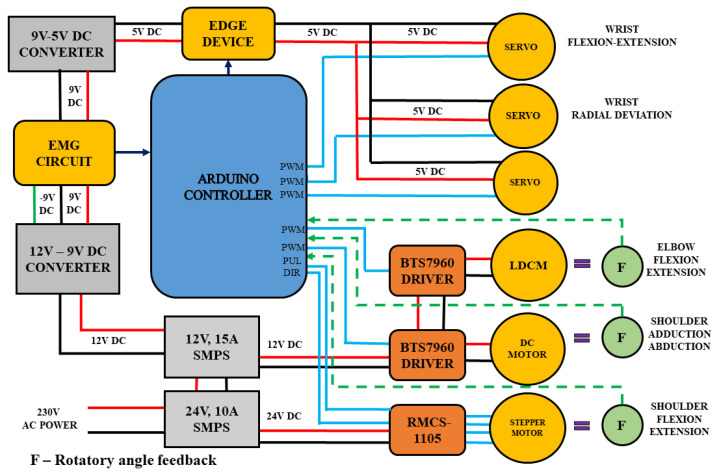
The design of the control unit and electrical wiring for the upper-limb mechanism and its driving system.

The range of motion planned for shoulder joint activity was set to 180° for flexion–extension and abduction–adduction actions individually, according to human physiology and anatomy [27]. A DC motor of 12 V and 30 kg/cm rated torque is employed to achieve abduction–adduction motion. The DC motor is fixed close to the seat of the wheelchair. A rod 8 mm in diameter and 60 cm long is connected to the DC motor shaft for holding the planetary geared stepper motor, NEMA34, which provides flexion–extension movements near to the patients’ shoulder. The 60 cm rod is fastened to the upper-limb frame with a round bearing. The rod is equipped with a gear, while another gear is employed with a rotatory angle feedback sensor. The motor aids in rotating the rod, and the attached gear provides the controller with an angular position. A stepper motor of 24 V 750 kg/cm rated torque is utilized to provide flexion–extension motion. The elbow and wrist structure of the Armeo spring is attached to the stepper motor by a gear. This mechanism is constructed as seen in Figure 2. A steel frame, attached to the wheelchair, is used to hold the DC motor and planetary geared stepper motor. The stepper motor holds the LDCM unit and aluminum frame to perform elbow joint actions. Figure 3 shows the details of the control unit design and electrical wiring for the upper-limb mechanism and its driving system. This is described in the following sub-section.

### 3.4. System Wiring

The lower-limb wiring is discussed elaborately in [24]. The upper-limb electrical wiring and control signals for actuators are described below. The control signals for corresponding physiotherapy routines are initiated by the edge device from the management console. These signals are sent to the Arduino controller through USB serial communication. A Pulse Width Modulation (PWM) signal is generated by the Arduino controller, which is used to actuate the wrist servo motor using 5 V DC power, generated by the buck converter. A set of three RKI-1203 servo motors are used for (i) flexion–extension, (ii) pronation–supination and (iii) radial–ulnar deviation. The servo motors, drawing a voltage of 5–8 V, are operated to a maximum of 3 A current drive, to provide a stall torque of 35 kg/cm. The LDCM motor for elbow flexion–extension and the DC motor for shoulder abduction–adduction are driven by BTS7960 driver boards, using a power source of 12 V and a 15 A current from a Switched Mode Power Supply (SMPS). The BTS7960 driver boards are activated by the PWM signal generated by the controller board. A rotatory angle feedback sensor is used to feed the position of angle movement to the controller. A stepper motor of 720 kg/cm rated torque is driven by the RMCS-1105 driver board. The pulse and direction signals from the controller are used to trigger the stepper motor for the flexion–extension movements. The stepper motor is activated by a 24 V, 10 A SMPS through an RMCS-1105 driver board. The rotatory angle feedback (F) potentiometer, as seen in Figure 3, is attached to the joints to give feedback signals to the controller.

## 4. Surface EMG Signal

**Figure 4:**
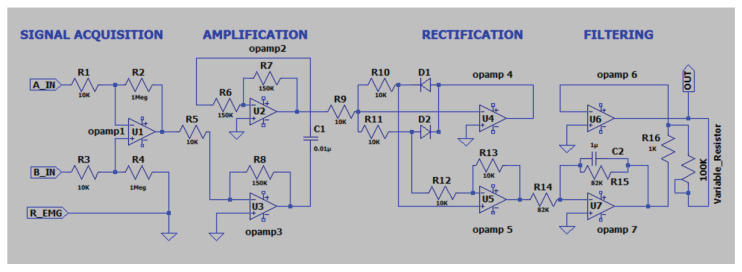
Layout of EMG signal-capturing circuit.

**Figure 5:**
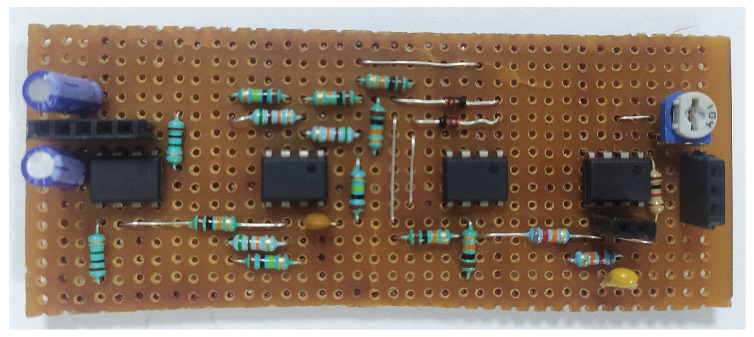
Snapshot of EMG circuit development.

**Figure 6:**
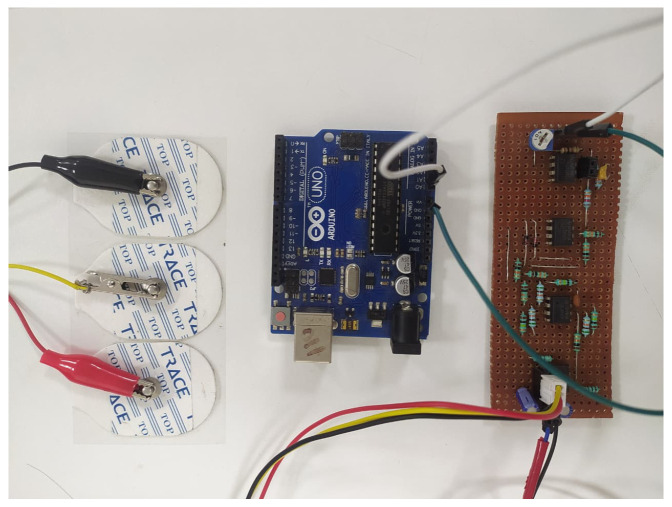
Photograph of EMG signal capturing circuit with 3 electrodes.

EMG signals are captured by surface electrodes placed on the muscles of the wrist, elbow, shoulder, backbone, leg and foot, in accordance with the SENIAM protocol [28]. The EMG signal recording process comprises four stages—(i) acquisition, (ii) amplification, (iii) rectification and (iv) filtering. A reference EMG *R_EMG* electrode is patched onto bone muscle, and two other electrodes, *A_IN* and *B_IN*, are fixed onto the targeted muscle movement area. The circuit is powered using a +9 V and −9 V DC power source from a 12 V SMPS through a buck converter. Signals *A_IN* and *B_IN* are fed into a differential amplifier gain of 110 to extract the voltage difference between them, and the targeted muscle signal is enhanced by suppressing common-mode noise and motion artifacts. The amplification stage uses two inverting operational amplifiers with a gain of 15 to boost the signal voltage by providing sufficient gain for processing. The rectification stage converts the amplified AC signal output to DC. The filtering process is used to reduce the ripple voltage in the circuit. A variable resistor of 100 kΩ is used to adjust the signal level at the output, as shown in Figure 4. Figure 5 and Figure 6 show photographs of the developed circuit.

## 5. Experiments and Results

**Figure 7:**
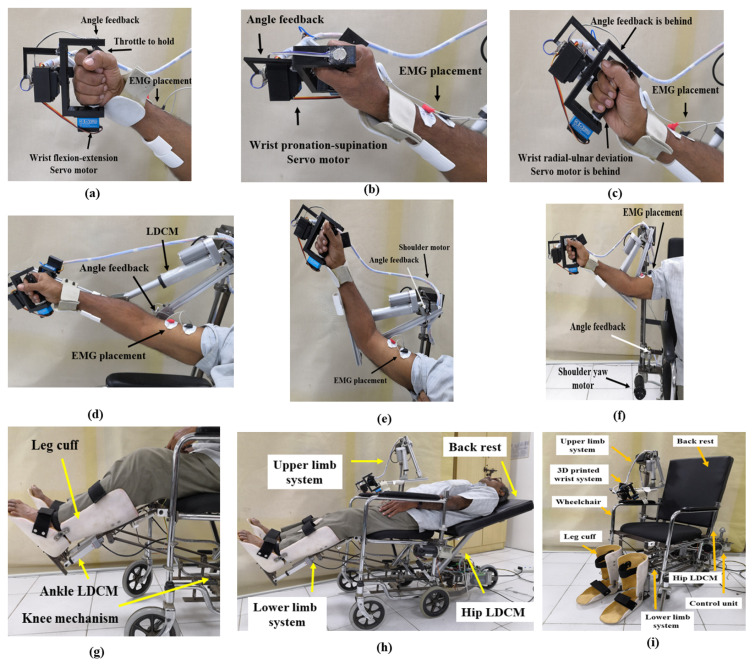
Photographs of wrist flexion–extension (**a**), wrist pronation–supination (**b**), wrist radial–ulnar deviation (**c**), elbow flexion–extension (**d**), shoulder flexion–extension (**e**), shoulder abduction–adduction (**f**), leg and ankle flexion–extension (**g**), hip rehabilitation (**h**), and overall system prototype (**i**).

**Figure 8:**
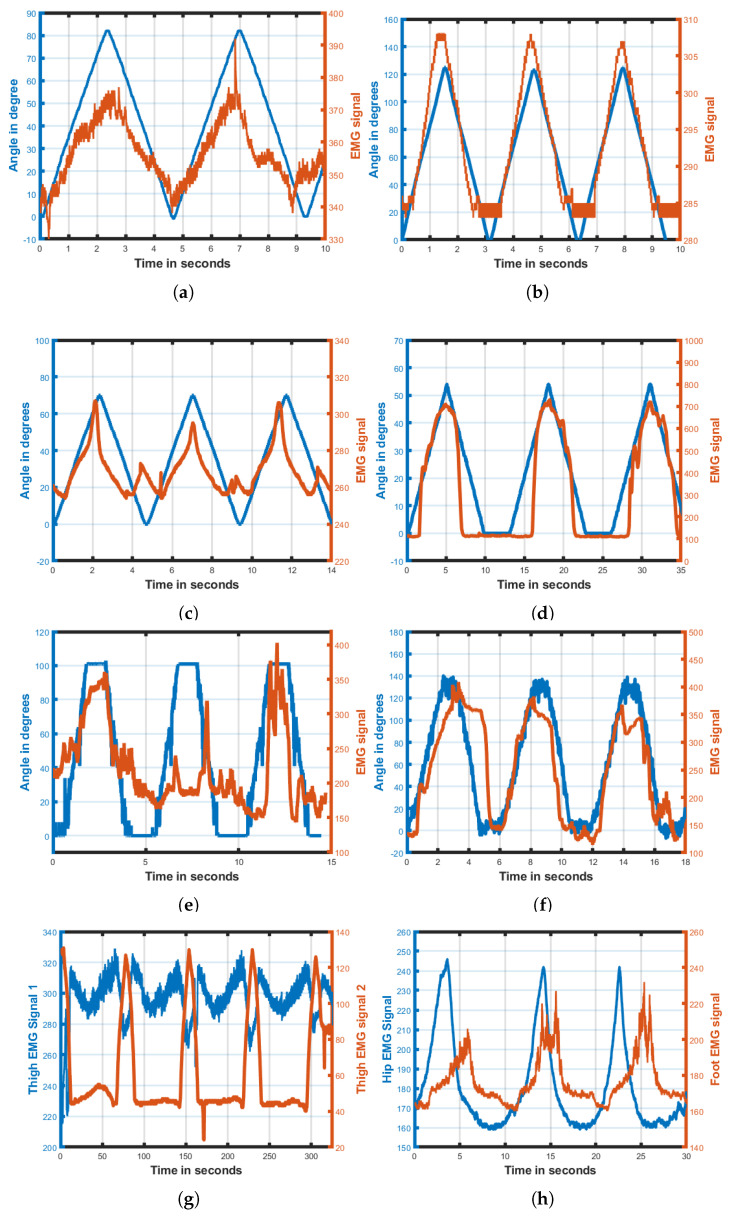
(**a**) Angle movement and EMG signals for wrist flexion–extension. (**b**) Angle movement and EMG signals for wrist pronation–supination. (**c**) Angle movement and EMG signals for wrist radial–ulnar deviation. (**d**) Angle movement and EMG signals for elbow flexion–extension. (**e**) Angle movement and EMG signals for shoulder flexion–extension. (**f**) Angle movement and EMG signals for shoulder abduction–adduction. (**g**) EMG signals for flexion–extension by placing both electrodes on top of thigh and another on adjacent side of thigh for knee flexion–extension. (**h**) EMG signal capturing for hip and ankle–foot flexion–extension.

The developed prototype of the upper- and lower-limb wheelchair-based rehabilitation device was experimentally verified. Figure 7 shows photographs of the system while in usage. The joint angle feedback from the device and EMG signals are captured and plotted in Figure 8. The system was applied with healthy control subjects to characterize the working of the proposed system. Necessary informed ethical consent was taken from all the control subjects with a declaration approving that the subjects had no prior neuromuscular weakness or injury. The data was collected in accordance with the Helsinki Declaration of 1975, as revised in 2000 [29]. The device performed passive rehabilitation with healthy control participants undergoing a range of exercise modes targeting the wrist, elbow, shoulder, knee and ankle joints without exerting any active effort. Joint angle feedback and surface EMG signals were simultaneously acquired to evaluate the coordination between muscle activity and joint movement during rehabilitation. A sampling rate of 1 kHz data was configured for recording rotatory angle feedback and EMG signals. These surface EMG signals acted as a reference and served as a target for each patient’s recovery. The range of motion (ROM) is defined as the limit to which a joint of the body can move. Column one of Table 2 lists various joint motions. The expected ROM values for the upper and lower limbs of a reference healthy person for different exercise modes are shown in column two of Table 2. Column three gives the measurements obtained from the developed system. The difference between the expected and actual system ROMs is shown in the last column. The results obtained for wrist flexion–extension, radial–ulnar deviation and shoulder abduction–adduction activity are promising, with less than 6.6% deviation. Figure 8 shows that the muscles are activated as per the angle of movement of the various exercise modes. The biceps brachii is a large, superficial muscle that is primarily responsible for elbow flexion, and it produces stronger and cleaner EMG signals compared to deeper muscles. In contrast, the thigh and shoulder muscles are often deeper and covered by thicker tissues, which can result in weaker or noisier EMG signals. The measured surface electrodes of the elbow, shoulder and thigh can be observed in Figure 8d,e,g. The smaller muscles around the wrist and ankle generate weaker and noisier EMG signals due to their limited muscle mass and deeper anatomical positioning, and can be observed in Figure 8a–c,h. The current tests were conducted only on healthy volunteers, which limits generalizability to patients with neurological disorders. Clinical trials involving stroke or similar patients with 34 participants [30], essential for assessing real therapeutic effectiveness, are yet to be performed. We are planning clinical trials to be conducted under expert supervision, focusing on patient safety and comfort. The trials will assess pain response, movement patterns and engagement with the device. The initial trials will begin with low-range movements, followed by a gradual increase in both range and exercise intensity.

## 6. Conclusions

In this work, the prototype model of a 9DoF system for upper- and lower-limb rehabilitation on a wheelchair, equipped with continuous EMG signal recording, is set up. The portable system is suitable for use in autonomous therapeutic rehabilitation routines for both the upper and lower limbs, and is targeted towards clinical and domestic usage. The system’s design, with its mechanical structures and electrical and control units, is described in this paper. The EMG signals acquired during joint movements aided by the proposed actuation system show muscle activation corresponding to the motions exercised. The prototype system supports subjects that are 4.8 to 5.8 feet tall and weigh up to 75 kg. The system is yet to be tested on actual stroke patients. Future work will focus on AI rehabilitation based on reinforcement learning (RL), where the system adapts therapy continuously by learning patient performance and provides a personalized and adaptive rehabilitation process that improves patient engagement, safety and recovery outcomes. We are exploring the use of dry or textile-based electrodes with adaptive filtering and high-resolution amplifiers to enhance signal quality and reduce noise. These approaches will support more reliable rehabilitation.

## Figures and Tables

**Table 1:** Survey of the literature on upper- and lower-limb-enabled rehabilitation devices.

Paper	IoT- Enabled	Exoskeleton Mechanism for Targeted Area							EMG Enhanced	Edge Communication	Cloud and Client Application
		Upper Limb				Lower Limb		Hip Flexion–Extension			
		3DoF Wrist	Elbow Flexion–Extension	Shoulder Flexion–Extension	Shoulder Abduction–Adduction	Knee Flexion–Extension	Ankle Flexion–Extension				
[10]	No	Yes	Yes	No	No	No	No	No	No	No	No
[11]	No	Yes	Yes	No	No	No	No	No	No	No	No
[12]	Yes	No	Yes	No	No	No	No	No	Yes	Yes	Yes
[13]	No	No	No	No	No	Yes	Yes	No	Yes	No	No
[14]	No	1 DoF	Yes	Yes	Yes	No	No	No	No	No	No
[18]	No	No	No	No	No	Yes	Yes	No	No	No	No
[20]	No	No	Yes	Yes	Yes	Yes	Yes	No	No	No	No
[24]	Yes	No	No	No	No	Yes	Yes	Yes	No	Yes	Yes
Proposed	Yes	Yes	Yes	Yes	Yes	Yes	Yes	Yes	Yes	Raspberry Pi	Yes

**Table 2:** Range of motion.

Exercise Mode	Total Expected ROM	Total Device ROM	Each ROM in Degrees	Difference
Wrist flexion and extension	78°	82°	0–40 flexion 40–82 extension	+4° (5%)
Wrist pronation and supination	180°	125°	0–60 pronation 60–125 supination	−55° (33%)
Wrist radial and ulnar deviation	66°	70°	0–30 radial 30–70 ulnar	+4° (6%)
Elbow flexion and extension	142°	55°	0–55 flexion 55–0 extension	−87° (53%)
Shoulder flexion and extension	180°	100°	0–100 flexion 100–0 extension	−80° (44%)
Shoulder abduction and adduction	150°	140°	0–140 abduction 140–0 adduction	+10° (6.6%)

## Data Availability

The original contributions presented in this study are included in the article. Further inquiries can be directed to the corresponding author.
